# Supplementary material for: Gate-tunable Veselago interference in a bipolar graphene microcavity
Source: Nat Commun. 2022 Nov 7;13:6711. doi: 10.1038/s41467-022-34347-w (PMC9640641; doi:10.1038/s41467-022-34347-w)
Supplement: Supplementary file 1 — Supplementary Information [file 41467_2022_34347_MOESM1_ESM.pdf]

Supplementary Information

Gate-tunable Veselago Interference in a Bipolar Graphene Microcavity

Xi Zhang[1†], Wei Ren[1†], Elliot Bell[1], Ziyan Zhu[2,3], Kan-Ting Tsai[1], Yujie Luo[4,5], Kenji Watanabe[6], Takashi Taniguchi[7], Efthimios Kaxiras[2,8], Mitchell Luskin[9], Ke Wang[1*]

[1]*School of Physics and Astronomy, University of Minnesota, Minneapolis, Minnesota 55414, USA*

[2]*Department of Physics, Harvard University, Cambridge, Massachusetts 02138, USA*

[3]*Stanford Institute for Materials and Energy Sciences, SLAC National Accelerator Laboratory, Menlo Park, California 94025, USA*

[4]*Department of Electrical and Computer Engineering, University of Minnesota, Minneapolis, Minnesota 55455, USA*

[5]*Department of Mechanical Engineering, University of Minnesota, Minneapolis, Minnesota 55455, USA*

[6]*Research Center for Functional Materials, National Institute for Materials Science, Tsukuba, Ibaraki, Japan*

[7]*International Center for Materials Nanoarchitectonics, National Institute for Materials Science, Tsukuba, Ibaraki, Japan*

[8]*John A. Paulson School of Engineering and Applied Sciences, Harvard University, Cambridge, Massachusetts 02138, USA*

[9]*School of Mathematics, University of Minnesota, Minneapolis, Minnesota 55414, USA*

Supplementary Note 1. Comparison of Two Fabrication Methods

As mentioned in Methods of the main text, we use two different bottom gate configurations to achieve the strain-induced microcavity wall electrostatics. One is to make adjacent bottom gates with a height difference, while the other is to use the silicon back gate and the local bottom gate to form a pn junction. For the first method, the advantage is that the fringing field would be more uniform and defined within the boundary of the cavity. As a result, the resonant peak positions for Device 3 matches with the theoretical expectation almost perfectly. However, the main disadvantage of this method is its difficulty in execution (device fabrication). For the gates that were made from two different e-beam lithography rounds, it is difficult to ensure that the edges of the two gates were perfectly parallel. This can give rise to a systematic variation of cavity width that contributes to additional broadening of the interference resistance peak. The low-yield and high requirement of the first method therefore make it unsuitable for scaling up the electron-optics devices that require more than one cavity

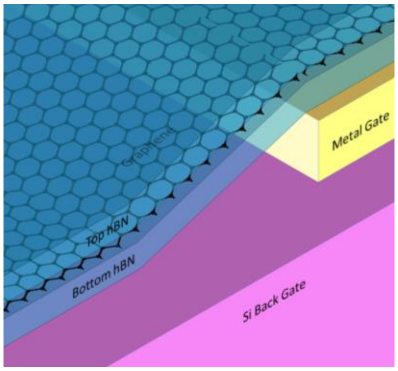

Supplementary Figure 1. Device scheme. Schematic image of the device architecture for Method 2. The electrostatics at two sides of the pn junction are controlled by the silicon back gate and local metal bottom gate, respectively.

(Device 1). For the second method, the fabrication is straightforward. The disadvantage is that, because of the 285 nm SiO_2 separation between the silicon back gate and the device, the spatial span of the fringing field is gate-dependent[1] while the position of the strain-defined cavity boundary is not. As a result, the pn junction can extend outside the cavity (between two strain-induced barriers). This could further break the electron-hole symmetry of the first- and second-order peaks and thus shift (slightly) the positions of the Veselago interference peaks. In addition to this, remote scattering from charge traps[2] in amorphous SiO_2 as well as larger surfaces roughness could also reduce the mean-free path of the electron in this region. However, this can be addressed by replacing the Si back gate with graphite gates for future devices.

Supplementary Note 2. Dependence of the Uniformity of Strain-induced Cavity Walls on Transfer Directions

Using the methods mentioned in the "Methods" section, we have fabricated multiple additional samples, with different transfer directions for comparison. Supplementary Fig. 2a-b show schematics of two cases of the sample dry-transfer process. The transfer directions of both Device 1 and Device 2 are along the gap between the gates (Supplementary Fig. 2a), while the transfer direction of Device 4 was perpendicular to the gap direction (Supplementary Fig. 2b). Supplementary Figure 2c shows the resistance as a function of the carrier densities of the p-doped and n-doped regions in Device 1. Different-order peak traces are labeled in the image, all of which cross at a single point. Supplementary Fig. 2d shows the resistance as a function of the carrier densities of the p-doped and n-doped regions in Device 4. Compared to the transport signals from Device 1 and Device 2 (main text fig.2b-c) where the Veselago interference is well defined for both first order and second order peaks, several sets of parallel peaks can be observed from Device 4. In Supplementary Fig. 2b, the fact that the trace lines (dashed lines) of the Veselago peaks converge at two different points indicates that two different-sized cavities

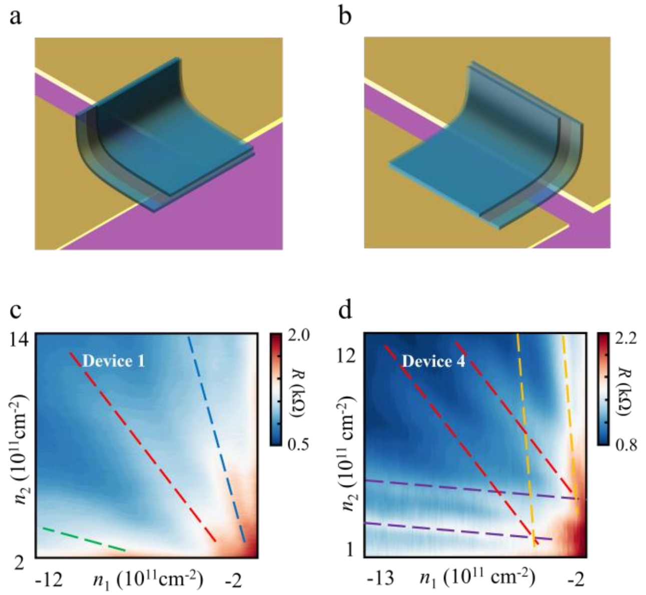

Supplementary Figure 2. Non-uniformed strain walls. (a) Schematic image of device during the transfer process of Device 1 and 2. (b) Schematic image of device during the transfer process of Device 4 and 5. (c) Resistance as a function of carrier densities of n-doped and p-doped region in Device 1. Different order peak traces are labeled, with crossing into one point. (d) Resistance as a function of carrier densities of n-doped and p-doped region in Device 4. First- and third-order Veselago interference peaks are labeled by red, purple, and yellow dashed lines. The two sets of peaks aggregate at two different points.

are formed simultaneously in Device 4. This shows that non-uniform strain-induced cavity walls are defined in Device 4. Since the non-uniform strain walls only appear at the junctions for which the transfer direction was perpendicular to the gap instead of along the gap direction, the emergence of another set of interference peaks may originate from the transfer direction during the sample preparation. As Supplementary Fig. 2a shows, when melting the sample on the gates, the transfer direction is along the gap direction, making it easier to keep the cavity width in Devices 1 and 2 uniform, as the pn junction boundary is also along the gap of the gates. However, for Device 4, the pn junction boundary direction is perpendicular to the transfer direction. In this case, to keep the cavity size constant, one needs to make sure the stack lands from one gated region to another gated region uniformly, which is typically difficult to control perfectly even with state-of-the-art dry transfer techniques.

Supplementary Note 3. Lower-bound Estimation of Carrier Mobility

Unlike a homogeneous Hall bar device, our device architecture is inhomogeneous by design with the presence of strain-defined cavity boundaries. A more quantitative estimation of the carrier mobility can therefore be less straightforward when using similar methods for Hall bar devices. We nevertheless performed experimental characterization of the lower-bound estimation of carrier mobility by two different methods and provided some elaboration on how to interpret the results in context of the more complicated device architecture.

Method 1: Estimation of carrier mobility using 4-probe resistance at zero magnetic field.

Unlike a Hall bar device, the cavity boundaries add significant resistance to the 4-probe R_{xx} at low field, making estimation of mobility using R_{xx} an underestimation of graphene quality. However, for a lower-bound estimation of mobility, we can still fit the Dirac peak from the data of pp regime (without being affected Veselago interference) by using the following formula[3]

$$\mathrm{R} = R_c + \frac{L}{W}\frac{1}{\mu e\sqrt{{n_0}^2 + \left(C_g\left|V_g - V_{Dirac}\right|\right)^2}},$$

where L and W are the length and width of the sample, e is the elementary charge, R_c is the contact resistance from graphene 1D contact, μ is the mobility, n_0 is the residual carrier density, C_g is the capacitance of the hBN per unit area, V_g is the gate voltage, and V_{Dirac} is the Dirac peak shift voltage. The mobility is estimated as ~ 90,000 $cm^2 V^{-1} s^{-1}$. Note that the fitting formula assumes a single uniform mobility (and conductivity) for the entire device, while multiple high-resistance and low-mobility strained boundary significantly contributes to the data are fitted. Therefore, we expect this to be a lower-bound estimation, with the actual device mobility significantly higher

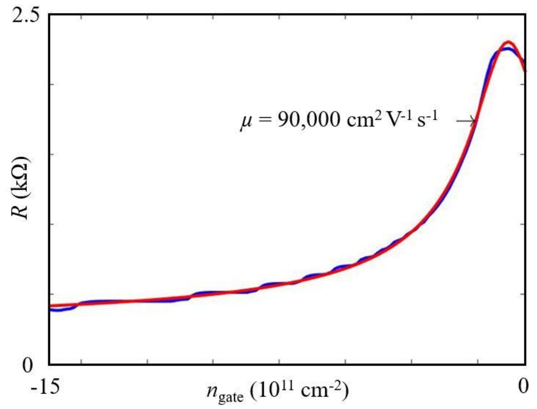

Supplementary Figure 3. Fitting of Resistance Versus Carrier Density. Blue curve is the original data of resistance as a function of carrier density, while the red curve is the fitting result. The mobility is characterized about 90,000 $cm^2 V^{-1} s^{-1}$.

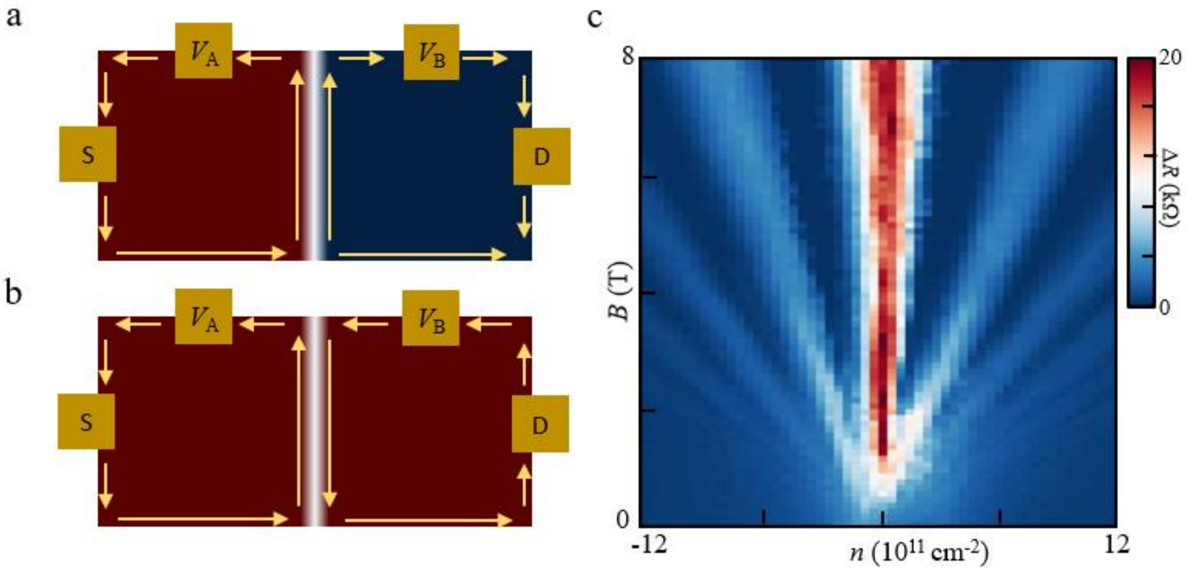

Supplementary Figure 4. Quantum Hall Edge States in pn and pp Junctions. (a)-(b) Locally-strained boundary can prevent the effective transmission of the quantum Hall edge states. (c) SdH oscillation can be observed starting at B = 1 T in a different device from the same stack.

than 90,000 cm^2 V^{-1} s^{-1} and closer to our previous rough estimation (~300, 000 cm^2 V^{-1} s^{-1}). However, the lower bound estimation of the mean-free path[4] of 0.9 µm, which is one order of magnitude larger than the cavity width, is sufficiently large to ensure uninterrupted interferences loops in the cavity.

Method 2: Using SdH oscillations or quantum Hall effect to estimate the carrier mobility.

Unlike Hall bar devices, the cavity boundary also prevents quantum Hall transport as the edge states becomes unequilibridized at the cavity boundary[5], making it difficult to accurately extract the mobility from SdH oscillations. This is why SdH oscillations are not observed in the micro-cavity device: even when the different regions of the sample are doped with the same carrier density, the locally strained boundary between gates can prevent the effective transmission of the quantum Hall edge states, and the transmission rate reduces as the magnetic field increases the effective gap at the strained boundary ($\Delta = \Delta_0 + \hbar\omega$). We have measured a different device region fabricated with exactly the same graphene stack (as the cavity studied in this work), but without strain and gate-defined boundaries. The SdH oscillations start to be visible at ~ 1T, giving an estimation of carrier mobility lower-bond of 90,000 cm^2 V^{-1} s^{-1}, or a mean-free path lower-bond[6] of 0.9 µm at the carrier density of 1.5×10^{12} cm^{-2} that is an order of magnitude longer than that of an interference loop.

Supplementary Note 4. Position of Veselago Peaks with Respect to Dirac Peak

If we extend the tracing line of each Veselago peak in our plots (see main text), they nearly cross at a single point in close-proximity to the Dirac peak, despite not being perfectly on top of it. This small discrepancy is due to the finite width of the strain-induced barrier that makes the carrier density on its two sides (the side (a) on the gate, and the side (b) in the cavity) slightly different.

Our figure shows the carrier density of the (a) side and is therefore slightly different than the carrier density at the boundary of the cavity on (b) side (which determines the Veselago physics in the cavity). In other words, when multiple Veselago interference peaks converge when the carrier density of side (b) approaches zero, our experimental parameter (the axis of our plots, corresponding to carrier density on the (a) side) is not yet strictly zero.

Supplementary Note 5. Field, Bias and Temperature Dependence of Additional Control Devices

Beyond Device 1, different orders of Veselago interference peaks are also observed in Devices 2, 4, and 5. As it was similarly presented in main text fig. 3d-f, the dependence of magnetic field, DC bias and temperature are shown in those control devices. In Supplementary Fig. 5a, we show the

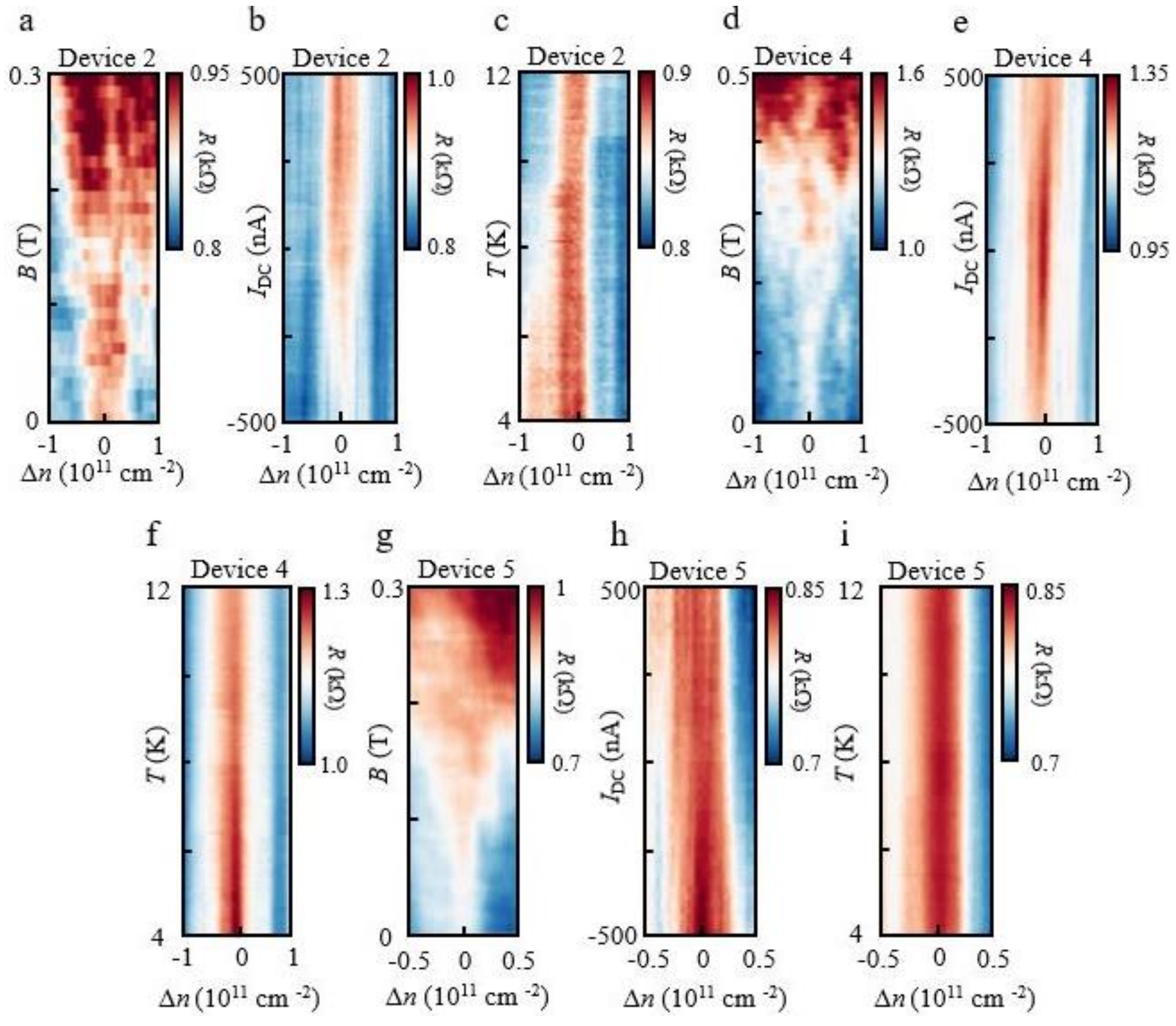

Supplementary Figure 5. Magnetic Field, Bias and Temperature Dependence of Control Devices. Measured resistance as a function of carrier density and magnetic field, bias and temperature around the peak position for Device 2 (a-c), Device 4 (d-f), and Device 5(g-i).

measured resistance of Device 2 as a function of magnetic field and carrier density around the peak position. The width of the interference peak monotonically increases with magnetic field, until it completely disappears. Supplementary Fig. 5b shows an asymmetrical bias dependence for second-order peaks at $|n_1| = 4|n_2|$. The asymmetrical bias dependence might come from the asymmetrical electrostatic distribution at the PN junction boundary, which shifts the charge neutrality point. As fig. 5c shows, there is no significant temperature dependence observed from 4K to 12K. While the first-order Veselago interference is destroyed with increasing magnetic field in Device 4 (Supplementary Fig. 5d), the bias and temperature scans show a weak dependence (Supplementary Fig. 5e, f) different from that of the first-order peak of Device 1 in fig.3e-f in the main text. This may be attributed to the uniformity of the strain-induced cavity wall of Device 4, such that the Veselago interference is more easily affected by Fermi surface tilting and thermal excitation. The dependence on magnetic field, bias, and temperature of the third-order peak of Device 5 are shown in Supplementary Fig. 5g-i. Like the data for the second-order peak of Device 2, slightly nonsymmetrical bias dependence is observed for third-order peaks at $|n_1|=9|n_2|$ and no significant temperature dependence is observed from 4K to 12K.

Supplementary Note 6. Peak Position (Resonant Condition) of the Veselago Interferences.
Consider a toy model for the resonant condition: we regard charge carriers, either electrons or holes, as semi-classical particles and approximate their trajectories as straight lines in both the p- and n-doped regions. This approximation is fairly accurate, as the carriers that manage to enter the cavity are those with small incident angles[7]. For the vast majority of the time along the carrier's trajectory in the cavity, the momentum projected to the axis perpendicular the cavity walls (k_y) dominates, and thus the approximation only starts to fail near the vicinity of the charge neutrality line. The distribution of carrier density n along the longitudinal direction (y-direction) is assumed to be linear, as expected from a uniform pn junction.

For the first order interference, we require the following condition to form a closed interference loop:

$$\left|\frac{k_{x1}}{k_{y1}}\right| = \left|\frac{k_{x2}}{k_{y2}}\right|,$$

where k_{x1} and k_{y1} are the wavevector of charge carriers in the transverse and longitudinal direction respectively in the p(n) region; k_{x2} and k_{y2} stand for the wavevectors in the n(p) region. Since there is no electric field in the x-direction, k_x should be conserved throughout the process:

$$k_{x1} = k_{x2}.$$

It then follows that

$$|k_{y1}| = |k_{y2}|.$$

Since most large-angle electrons are reflected by the cavity wall before entering the cavity, we

only consider carriers with small incident angle into the cavity, expressed as

$$k_x \ll k_y,$$

where k_y can be approximated as

$$k_y = \sqrt{k_F(n)^2 - {k_x}^2} \approx k_F(n).$$

In monolayer graphene, the relationship between the carrier density n and k_F is

$$k_F(n) \propto \sqrt{|n|}.$$

Then we can obtain the resonance condition of the first order interference

$$|n_1| = |n_2|.$$

To form a second order interference loop, it relation of n_1 and n_2 can be written as:

$$|n_1| = t|n_2|$$

Then the ratio between k_y is:

$$|k_{y1}| = \sqrt{t}|k_{y2}|.$$

As k_x component does not change because of zero displacement field along x-direction, the relation between the incident angles θ_1 and θ_2 at p-side and n-side is:

$$\frac{\tan\theta_1}{\tan\theta_2} = \frac{\left|\frac{k_{x1}}{k_{y1}}\right|}{\left|\frac{k_{x2}}{k_{y2}}\right|} = \left|\frac{k_{y2}}{k_{y1}}\right| = \frac{1}{\sqrt{t}}$$

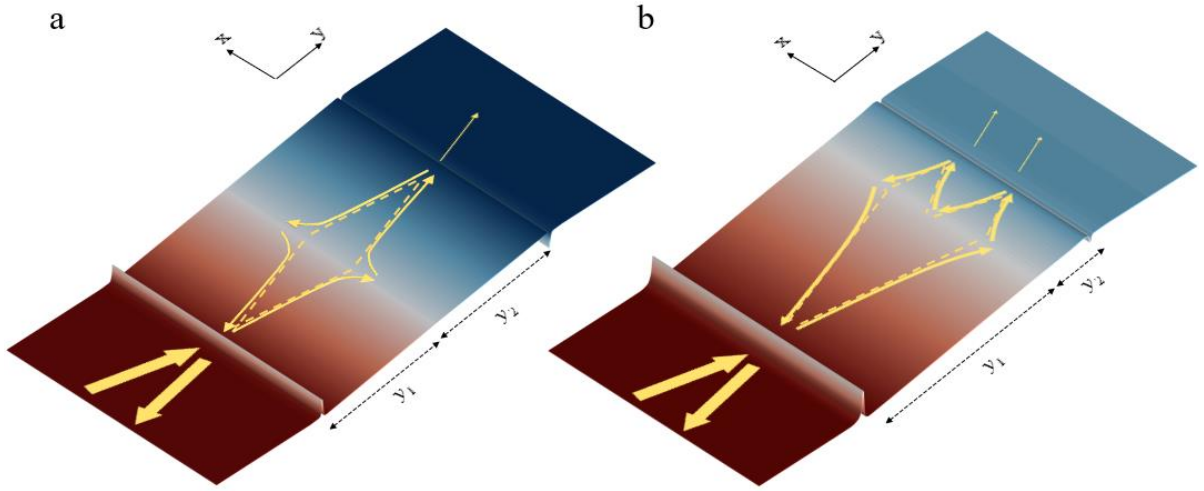

Supplementary Figure 6. Schematics of (a) first- and (b) second-order interference processes. Dashed lines represent an approximated path of electrons (holes).

At the same time, according to the geometric relation, it has:

$$\frac{\tan\theta_1}{\tan\theta_2} = \frac{2}{t}$$

as the charge carriers bounces two times at n_2 side boundary, and the ratio of the length of the p-n region in the cavity equals to t when the carrier density varies linearly in the cavity. Therefore, the parameter t can be solved as $t = 4$.

Thus

$$|k_{y1}| = 2|k_{y2}|.$$

and

$$|n_1| = 4|n_2|.$$

Similarly, $4|n_1| = |n_2|$ is the resonant condition of forming the other second order interference path. Other higher order resonant conditions, $|n_1| = 4|n_2|$, $9|n_1| = |n_2|$, and so forth, can be achieved with a similar argument.

Supplementary Note 7. Slight Modification of Resonant Condition Due to Pseudo Barrier

Now consider the fact that k_x is finite, which results in imaginary k_y near the charge neutrality line. This small but finite region (Supplementary Fig. 7) with an imaginary k_y is where tunneling happens and the electron wavefunction exponentially decays, referred to here as the pseudo-barrier. It is noted that the width of this region, w, also depends on k_x. The higher k_x gives rise to larger w.

For the first order interference, the resonance condition is

$$|n_1| - \frac{w}{2} = |n_2| - \frac{w}{2},$$

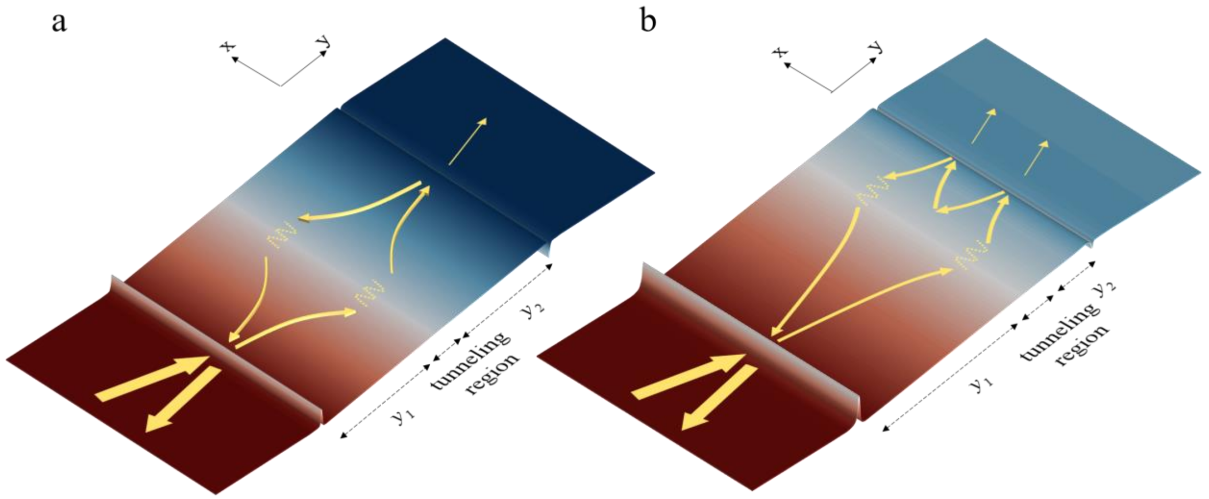

Supplementary Figure 7. Schematic of (a) first- and (b) second-order interference processes including the finite pseudo-barrier near the charge neutrality line.

which again requires

$$|n_1| = |n_2|.$$

For the second order interference, the resonance condition is modified to

$$|n_1| - \frac{w}{2} = 4\left(|n_2| - \frac{w}{2}\right)$$

$$\Rightarrow |n_1| = 4\,|n_2| - 2w < 4|n_2|.$$

The resonance condition thus occurs slightly towards $|n_1| = 3|n_2|$. Besides, since different k_x values correspond to different w, a certain distribution in k_x will result in the broadening of resonance condition. Similar arguments can be applied with other higher order resonant conditions.

Supplementary Note 8. Simulation of Charge Carrier Trajectory in Linearly Graded PN-junction at Zero Magnetic Field

Following the coordinate system as drawn in Supplementary Fig. 8a, we can set the origin on the charge neutrality line, with x-axis parallel to the charge neutrality line. Then the carrier density distribution inside the cavity can be expressed as

$$n(y) = -\frac{n_1}{d_1}y = \frac{n_2}{d_2}y,$$

where $d_1(d_2)$ is the distance between the charge neutrality line and the lower(upper) cavity wall, $n_1(n_2)$ is the carrier density on the p (n) side outside the cavity. By summing over all occupied states below the Fermi surface, $n(y)$ can also be written as

$$n(y) = 4 \cdot \frac{1}{L_1L_2}\int_0^{k_F(y)} \frac{2\pi k\, dk}{4\pi^2/L_1L_2} = \frac{1}{\pi}\, {k_F}^2(y).$$

The magnitude of wavevector at the Fermi surface $k_F(y)$ is therefore given by

$$k_F(y) = \sqrt{\pi n(y)}.$$

Considering the carrier density is uniform along the x direction, the x component of the wavevector should be conserved:

$$k_x(y) = k_x(y = -d_1) = k_1 \sin\theta_1 = \sqrt{\pi n_1}\sin\theta,$$

where k_1 is the magnitude of wavevector of the incident charge carrier and θ is its incident angle into the microcavity. In the last step we have used $k_1 = k_F(y = -d_1) = \sqrt{\pi n_1}$. Thus

$$k_y(y) = \sqrt{{k_F}^2(y) - {k_x}^2(y)} = \sqrt{\pi n(y) - \pi n_1 \sin^2\theta}.$$

From the dispersion relationship

$$E = \hbar v_F\sqrt{{k_x}^2 + {k_y}^2},$$

we can obtain the x and y components of group velocity of the charge carrier

$$v_x = \frac{1}{\hbar}\frac{\partial E}{\partial k_x} = \frac{k_x v_F}{\sqrt{{k_x}^2 + {k_y}^2}},$$

$$v_y = \frac{1}{\hbar}\frac{\partial E}{\partial k_y} = \frac{k_y v_F}{\sqrt{{k_x}^2 + {k_y}^2}}.$$

Then we have

$$\frac{dx}{dy} = \frac{v_x}{v_y} = \frac{k_x}{k_y} = \frac{\sqrt{\pi n_1}\sin\theta}{\sqrt{\pi n(y) - \pi n_1 \sin^2\theta}}.$$

Substituting in the expression of $n(y)$ corresponding to a linearly varying density pn-junction, the differential equation of the electron trajectory yields

$$dx = \frac{\sqrt{\pi n_1}\sin\theta}{\sqrt{-\pi \frac{n_1}{d_1} y - \pi n_1 \sin^2\theta}} dy$$

Considering a charge carrier injected from the p side of the micro-cavity, we notice that y should satisfy

$$y < -d_1 \sin^2\theta.$$

Further from $y = -d_1 \sin^2\theta$, k_y will become imaginary and the electron will enter the tunneling region. Integral over the differential trajectory equation before the electron enters the tunneling region, it yields

$$\int_0^x dx = \int_{-d_1}^{y} \frac{1}{\sqrt{-\frac{1}{d_1 \sin^2\theta} y - 1}} dy = \int_{-d_1}^{y} \frac{1}{\sqrt{ay-1}} dy,$$

$$\Rightarrow x = \frac{2\sqrt{ay-1}}{a} - \frac{2\sqrt{-ad_1 - 1}}{a} = \frac{2\sqrt{ay-1}}{a} - b,$$

where $a = -\frac{1}{d_1 \sin^2\theta}$ and $b = \frac{2\sqrt{-ad_1-1}}{a} = -2d_1\sqrt{\sin^2\theta\,(1-\sin^2\theta)}$. After simplification, we find

$$y = \frac{a}{4}(x+b)^2 + \frac{1}{a}.$$

Similarly, the trajectory of charge carrier above the charge neutrality line can be derived.

Based on the trajectory equation, we will show that the condition to form a second order interference loop is $|k_{y1}| = 2|k_{y1}|$ (or $|n_1| = 4|n_2|$) in the following step.

Supplementary Fig. 8b provides a schematic of charge carrier trajectory inside the cavity when the carrier undergoes two Veselago refractions and one (three) reflection at $|n_1| = t|n_2|$ with t be a real number. 'A' is the starting point of a charge carrier right after it enters the cavity; 'B' is the farthest point the carrier can travel in the p side of the cavity and is where it starts to enter the

tunneling region; 'C' is the position of carrier right after it tunnels into the n side; 'D' is where the carrier gets reflected by the cavity wall on the n side. The coordinates of these points can be calculated from the above trajectory equation:

$$A\colon\left(0, \frac{ab^2}{4} + \frac{1}{a}\right),$$

$$B\colon\left(-b, +\frac{1}{a}\right),$$

$$C\colon\left(-b, -\frac{1}{a}\right),$$

$$D\colon\left(\sqrt{-4\frac{ad_2+1}{a^2}} - b, d_2\right).$$

Thus,

$$x_{AB} = -b,$$

$$x_{CD} = \sqrt{-4\frac{ad_2+1}{a^2}}$$

Geometrically, it must satisfy the following condition for the charge carrier to come back to 'A' after two Veselago refractions and one reflection (or a first order Veselago interference loop to form) as in Supplementary Fig. 8a:

$$x_{AB} = x_{CD}.$$

After simplification, it requires

$$d_1 = d_2.$$

which equivalently requires a specific ratio of the carrier densities on two sides of the cavity:

$$|n_1| = 4|n_2|.$$

Similarly, it must satisfy the following condition for the charge carrier to come back to 'A' after two Veselago refractions and three reflections (or a second order Veselago interference loop to form) as in Supplementary Fig. 8b:

$$2x_{AB} = 4x_{CD}.$$

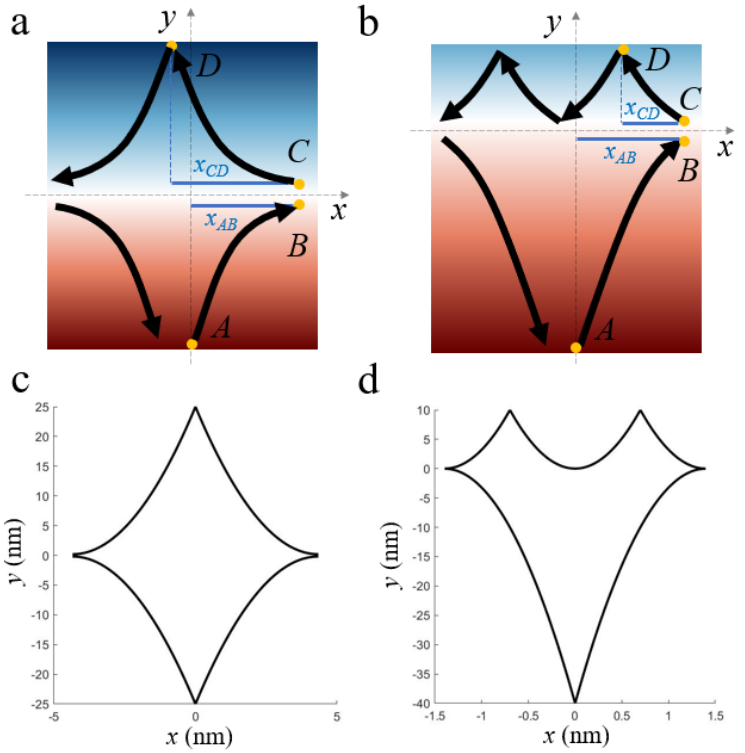

Supplementary Figure 8. Simulation on Charge Carrier Trajectory when Second-order Veselago Interference Peak Developed. (a)-(b) Schematics of charge carrier trajectory when it undergoes two Veselago refractions and one (three) reflection. A closed first (second) order interference loop can be formed only when $x_{AB} = x_{CD}$ $(2x_{AB} = 4x_{CD})$. (c)-(d) Simulated close-loop trajectories at resonant condition (c) $|n_1| = |n_2|$ and

After simplification, it requires

$$(1 + 3\sin^2\theta)d_1 = 4d_2.$$

When θ is small, it reduces to

$$d_1 = 4d_2,$$

which equivalently requires a specific ratio of the carrier densities on two sides of the cavity:

$$|n_1| = 4|n_2|.$$

Supplementary Fig. 8c (8d) shows the simulation result when $|n_1| = |n_2|$ ($|n_1| = 4|n_2|$) with the incident angle $\theta_1 = 5°$. A closed loop can form when the first (second) order resonant condition is satisfied.

Supplementary Note 9. Width and DC Bias Dependence of 2nd Order Veselago Interference Peaks

The edge of the gates can have a roughness on the order of several nanometers during lift-off. As a result, the distance and effective angle between two cavity walls varies slightly. Depending on where the carriers are injected, the size and shape of full-cycle interference loops (along with the resonant condition) may vary slightly, leading to a broadening of the resonant condition around its theoretically-expected value ($|n_1| = |n_2|$ for first-order peak). The schematics of the cavity with rough walls and the interference paths of charge carriers are shown in Supplementary Fig. 9.

Higher-order interference peaks exhibit less broadening. Here we provide a qualitative argument. For the second-order peak, charges are injected at a small incident angle via the cavity wall at which high carrier density is found. The Fermi wavelength here is comparable to the roughness of cavity, resulting in more uncertainty in its effective injection angle (diffusive injection). However, the broadening of the injection angle distribution does not directly contribute to the broadening of the resonant peak, since the interference loop can be formed at the same resonant condition ($|n_1| = 4|n_2|$) independent of specific incident angle, as previously elaborated. For the two consecutive reflections against the other boundary that could contribute to the broadening (if the reflection were diffusive), the carrier density is significantly lower (by a factor

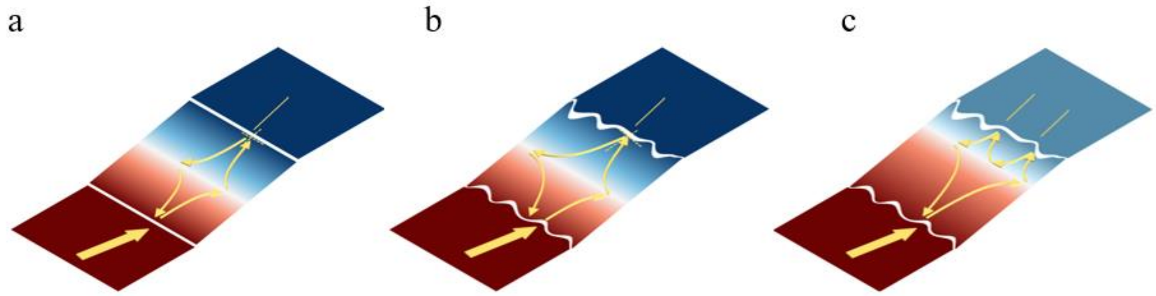

Supplementary Figure 9. Schematics of the first-order interference process in a cavity with (a) perfectly straight walls and (b) rough walls. Charge carriers are more likely to experience diffusive reflection at the second cavity wall and thus cannot form a closed interference loop as if the walls were straight. (c) Schematics of second-order interference in a cavity with rough walls. The charge carriers are less sensitive to the roughness of the second wall due to their larger fermi wavelength.

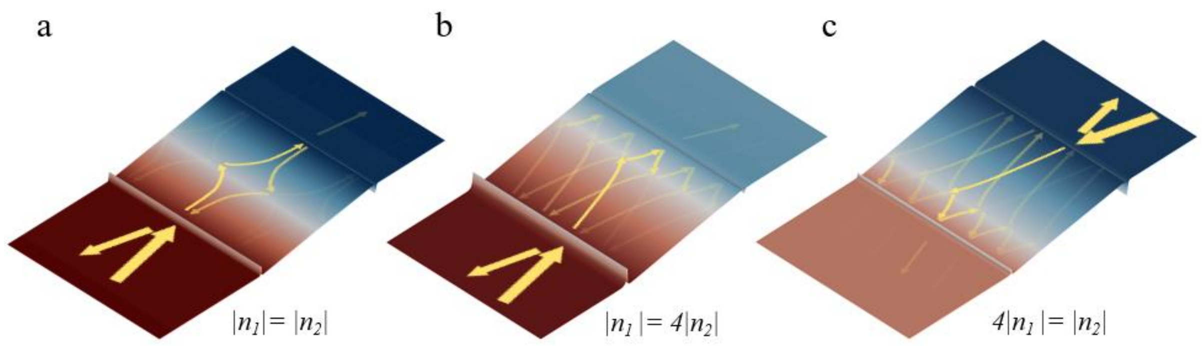

Supplementary Figure 10. More accurate schematics of the first- and second- order interference loops. The transparency of arrows inside the cavity indicates the number of charge carriers along the path. (a) The main interference loop still dominates when $|n_1| = |n_2|$. (b-c) The side loops play a larger role during the interference when $|n_1| = 4|n_2|$ and $4|n_1| = |n_2|$.

of 4 for the second-order peak, comparing to the first-order peak) and the Fermi wavelength is significantly larger, making it less sensitive to the roughness of the cavity wall, and thus less likely to result in diffusive reflections as shown in Supplementary Fig. 9c. Following the same logic, the third-order peak should be even sharper, which agrees well with our experimental observation.

The cartoon in figure 4a shows one particular bias direction where carriers are injected through the cavity wall closest to the source, where electrons with finite incident angles are allowed into the cavity with small Klein-tunneling probability. The injected electrons and their angle-distribution are the same for $|n_1| = 4|n_2|$ and $4|n_1| = |n_2|$ as it is via the strain-defined Klein barrier, independent of electrostatics. However, when the same electron reaches the opposite cavity wall, the incident angle is larger (smaller) for $4|n_1| = |n_2|$ ($|n_1| = 4|n_2|$) due to lower (higher) doping compared to where it is injected. This is what "breaks" the symmetry of the interference loops illustrated in our cartoons where the bias direction is kept the same for the ease of discussion. During the major part of the experiment, an AC bias is applied on top of a zero DC offset, and $|n_1| = 4|n_2|$ and $4|n_1| = |n_2|$ is equivalent. The only exception is figure 4e where a DC bias is intentionally applied to observe the asymmetry expected from the narrative above.

Supplementary Note 10. Full Interference Paths in the Cavity

In the main manuscript, for the ease of discussion and essential physics, we have only considered charge carriers that successfully form the desired interference loop back to their original positions on via the most direct path. Here, we show that for the rest of the carriers, which undergo a more complicated trajectory inside the cavity, they eventually form an interference loop at the same resonant condition as well. Supplementary Fig. 10a-c provides a more accurate description of the interference loops, including the higher order trajectories when $|n_1| = |n_2|$, $|n_1| = 4|n_2|$, and $4|n_1| = |n_2|$, respectively. The transparency of arrows inside the cavity indicates the number of charge carriers moving along the path. We consider that the reflection rate of cavity walls is high[8] (> 50%, since the tunneling rate decays quickly with increased incident angle) and that the transmission rate at the charge neutrality boundary is high (> 50%, due to the fact that only charge carriers with

small incident angle can get into the micro-cavity)[9,10]. Additionally, we consider charge carriers with larger incident angle to have a higher possibility of being reflected either by the cavity walls or at the charge neutrality boundary. In Supplementary Fig. 10a, the main interference loop still dominates the side loops; in Supplementary Fig. 10b-c, the main interference loop is not as dominant as in Supplementary Fig. 10a, while the net effect of all interference paths is that more carriers are either trapped inside the cavity or reflected to the source side. Veselago interference thus helps further collimate the charge carriers.

Supplementary Note 11. Enhanced Collimation from Second-order Veselago Interference

Veselago interference can be used to further collimate carriers, particularly those with small incident angles. In Supplementary Fig. 11, device diagrams show that source (drain) contacts are intentionally placed at the bottom right (left) corners of graphene and two 1μm-wide voltage probes A and B are placed at the top and bottom edges of the graphene near the drain side. When $|n_1| < |n_2|$ (Supplementary Fig. 11a), the uncollimated carriers are injected from the injection point at the source with different incident angles. As the doping at the source side (region before entering the cavity) is higher than the doping at drain side (region after passing the cavity), the incident angle of a carrier that goes into the cavity is supposed to be smaller than the refraction angle of the same carrier when after it passes the cavity. Then, the carriers will refocus at the focus point which is highlighted as the red spot before reaching at the drain. In this case, carriers can accumulate at probe B and the measured transverse voltage is proportional to the current density from uncollimated charge carriers. At $4|n_1| = |n_2|$, Veselago interference will further localize uncollimated carriers in the cavity, resulting in a near-zero measured transverse voltage. Similarly, when $|n_1| > |n_2|$ (Supplementary Fig. 11b), the incident angle of a carrier that goes into the cavity is supposed to be larger than the refraction angle of the same carrier when after it passes the cavity. Starting from the injection at the source, carriers move to the focus point after passing the cavity. However, as the focus point is supposed to be further away from the cavity than the drain, carriers will reach the physical edges of the sample first, resulting in the random scattering of carriers (labeled as the red curved arrows). Therefore, the measured voltage

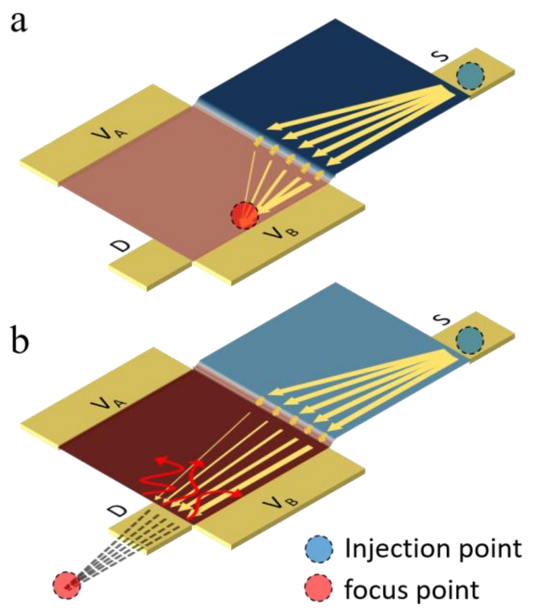

Supplementary Figure 11. Electron Trajectories Schemes of Second-order Veselago Interference. (a) When $|n_1| < |n_2|$, carriers are injected with different incident angles from the injection point at one side of the junction, then refocus at the focus point at the other side of the junction. (b) When $|n_1| \geq |n_2|$, carriers will reach the sample physical edge instead of reaching the focus point.

difference across the probe A and probe B is not eligible for collimation characterization anymore.

Supplementary Note 12. Decoherence of Veselago Interference Peaks

Aside from the Veselago interference peaks, we did not observe other oscillatory behavior (as a function of k) as an implication of spatial phase. We attribute this to the decoherence between distribution of different interference loops and realistic inhomogeneities of strain-defined barrier, which we elaborate below.

The path of a specific Veselago interference loop L (in a semi-classical picture), is not strictly well-defined near the charge neutrality point, where k becomes imaginary (tunneling) and electron wavelength becomes larger than the cavity. Quantitative theoretical model goes beyond the scope of this work. However, at the limit of small incident angle incidence, the effective tunnel region is narrow and semi-classical picture can be used as a qualitative approximation.

Due to collimation efficiency of the strain-induced barrier, only carriers with small incident angle can enter the cavity, with angle distribution of

$$I(\theta) = A(\theta)e^{-i0} = A(\theta) = A_0 G(\theta) = \frac{A_0}{\sigma\sqrt{2\pi}} e^{-\frac{\theta^2}{2\sigma^2}},$$

where A_0 is a constant proportional to the applied current, σ is a small angle-distribution, depending on the device specifics such as strain-induced barrier height. For any given data point, a distribution of interference loops with different incident angles contributes simultaneously to the observed Veselago interference peak (Supplementary Fig. 12a), each with different spatial phase.

For the interference loop with incident angle θ, the Klein tunneling probability at the charge neutrality point can be approximated as

$$T(\theta) = T_0 e^{-\gamma\theta^2},$$

where T_0 is the normalization constant and γ is a constant. The angle distribution of the charge carriers forming the Veselago interference loop in the cavity can be approximated to be:

$$P(\theta) = I(\theta)T^2(\theta) = \frac{A_0 {T_0}^2}{\sigma\sqrt{2\pi}} e^{-\frac{\theta^2}{2\sigma^2} - 2\gamma\theta^2}.$$

Assuming the cavity width d is constant (~ 50 nm) in the device, the electron wavefunction interferes with itself after accumulating spatial phase with itself. The interference amplitude from a loop with incident angle θ is given by

$$\left| \frac{1 + t e^{-i\phi_V(k,\theta)}}{\sqrt{1+t^2}} \right|^2 = 1 + \frac{2t}{1+t^2} \cos\phi_V(k,\theta),$$

where t is a constant. Where the spatial phase $\phi_V(k,\theta)$ can be written as

$$\phi_V(k,\theta) = 4\int_{-\frac{d}{2}}^{\frac{1}{a}} k_x(y)\frac{dx}{dy}dy + 4\int_{-\frac{d}{2}}^{\frac{1}{a}} k_y(y)\,dy$$

$$= 4\int_{-\frac{d}{2}}^{\frac{1}{a}} k\sin\theta\sqrt{\frac{1}{-a}(-y+\frac{1}{a})}^{-\frac{1}{2}}dy + 4\int_{-\frac{d}{2}}^{\frac{1}{a}} k\sqrt{-\frac{2}{d}y-\sin^2\theta\,dy}$$

$$= 4kd\sin^2\theta\,(1-\sin^2\theta)^{\frac{1}{2}} + \frac{4}{3}kd(1-\sin^2\theta)^{\frac{3}{2}} \approx \frac{4}{3}kd + 2kd\theta^2 + O(\theta^4),$$

where $a = -\frac{2}{d\sin^2\theta}$, $b = \frac{2\sqrt{-ad/2-1}}{a}$, $k_x(y) = \sqrt{\pi n}\sin\theta$ and $k_y(y) = \sqrt{\pi n(y) - \pi n\sin^2\theta}$ with $n(y)$ and n defined as the carrier density inside the cavity and the carrier density outside the cavity, respectively (as defined in Supplementary Note 8).

The measured resistance R, is proportional to the interference amplitude contributed from all participating interference loops (summing over θ):

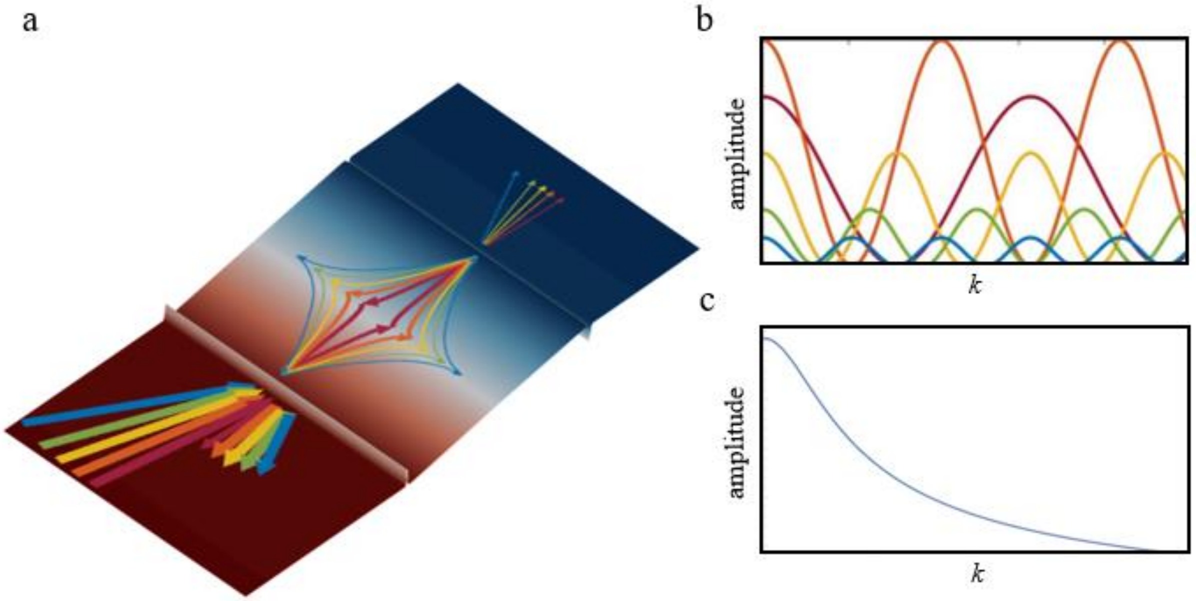

Supplementary Figure 12. Interference Paths in the Microcavity. (a) Simulations demonstrate the carrier trajectories in the micro-cavity when the incident angles are 1° (red), 2° (orange), 3° (yellow), 4° (green), 5° (blue), respectively. Different incident angles can bring significant changes in the interference paths and *kL* phases. (b) Illustration of interference amplitude corresponding to each path in (a) as a function of incident *k* with distinct oscillation frequency. (c) Calculated result of the interference amplitude (if disregarding the $\frac{4}{3}kd$ term during the summation, which is not observed from the data) from all paths (not limited to this five) as a function of incident wave vector *k*. The *k* dependence is exponential decaying.

$$A(k) = \int_{-\frac{\pi}{2}}^{\frac{\pi}{2}} P(\theta)\left(1+\frac{2t}{1+t^2}\cos\phi_V(k,\theta)\right)d\theta$$

$$= \int_{-\frac{\pi}{2}}^{\frac{\pi}{2}} \frac{A_0{T_0}^2}{\sigma\sqrt{2\pi}} e^{-\frac{\theta^2}{2\sigma^2}-2\gamma\theta^2}\left(1+\frac{2t}{1+t^2}\cos\phi_V(k,\theta)\right)d\theta.$$

We discuss the implication of phase $\phi_V(k,\theta)$ after the integration:

1. The $2kd\theta^2$ term captures the decoherence between interference loops. As an intuitive example, Supplementary Fig. 12b individually plots the calculated Veselago Interference resistance from five different interference loops simulated in Supplementary Fig. 12a. Despite the oscillatory behavior expected from each interference loop, the oscillation periodicity depends sensitively on incident angle. The measured resistance R at the Veselago interference peaks, is a summation of contribution from all co-existing interference loops (instead of summation over k), which (Supplementary Fig. 12c) shows exponential-decaying profile of k (instead of oscillatory).
2. The $\frac{4}{3}kd$ term predicts a slowly-oscillating behavior on top of the fast-decaying profile, under the conditions of well-defined and comparably large d. None of the conditions is applicable in our device architecture, cross-checked by multiple characterizations within our experiment. Specifically, (1) With the estimated cavity width of ~50 nm, the expected oscillation periodicity is comparable to the entire carrier density range of the measurement (10^{12} cm^{-2}). (2) Realistic inhomogeneity along strain-defined cavity boundary (location, effective width and height) exists, as characterized by multiple different devices (Supplementary Note 5). The inhomogeneity leads to decoherence of the kd term while keep the Veselago interference intact. For example, Veselago interference condition stays independent of d, while spatial phase kd sensitively depends on it. The absence of Fabry-Perot interference (as a direct prediction of kd phase) has also been experimentally verified at the PP and NN regime of multiple devices (fig. 1d).

The lack of overall coherence of Veselago interference is also consistent with the temperature dependence. The Veselago resistance peak height is fairly unaffected by the implication of thermal fluctuation (Supplementary Fig. 5).

Supplementary Note 13. Qualitative Difference Between Veselago interference and Fabry-Perot Interference

There are three experimental observations that led us to believe the observation of resistance peaks cannot be attributed to F-P.

First, the Veselago interference requires a close loop interference path in the cavity, that exists

only when a pn junction is defined in the cavity, which agrees with our experimental observation. The parallel paths of F-P interference, in contrast, should in-principle exist for all combinations of carrier types (pp, nn, pn, np), as the two strain-induced barriers exist independently. The fact we see the resistance peaks only in the pn or np regime implies that the observation is a consequence of Veselago interference instead of F-P interference.

Second, the location at which F-P interference peaks are found as a function of carrier densities should be in the shape of interference fridges that depend on electron wave vector k, instead of when resonance conditions are met ($|n_1| = |n_2|$, $4|n_1| = |n_2|$ or $|n_1| = 4|n_2|$). The position of observed resistance peaks therefore cannot be explained by the F-P mechanism even at a qualitative level.

Third, the novel device architecture is designed for Veselago interference, instead of F-P. The central cavity region containing the interference paths is in-between two gates, instead of on top of another central gate. The carrier density in the cavity smoothly changes (as a part of experimental design), instead of being homogeneously defined. We therefore do not expect the F-P interference paths to be effectively defined, in a way similar to that of previous work on pnp or npn junctions.

Supplementary Note 14. Resistance of strain-induced barrier

When biased at pp or nn region (in absence of pn junctions), the 4-probe resistance of the device can be written as $R_{tot} = R_G + 2R_{cw}$, where R_G is the graphene resistance, R_{cw} is average resistance from a single cavity wall. At high dopings (when $n_1 \sim n_2 \sim 10^{12}$ cm^{-2}), R_{tot} of the cavity device is measured to be ~ 0.32 kΩ. At the same doping, R_G is estimated to be ~ 0.01 kΩ, from measuring a different region of the same graphene stack without any strain-induced or electrostatically defined barrier. This gives an estimation of R_{CW} ~ 0.155 kΩ, similar to that of a Klein barrier defined by a pn-junction with known ineffective collimation (or finite Klein angle-dependence) preferred by our device architecture.

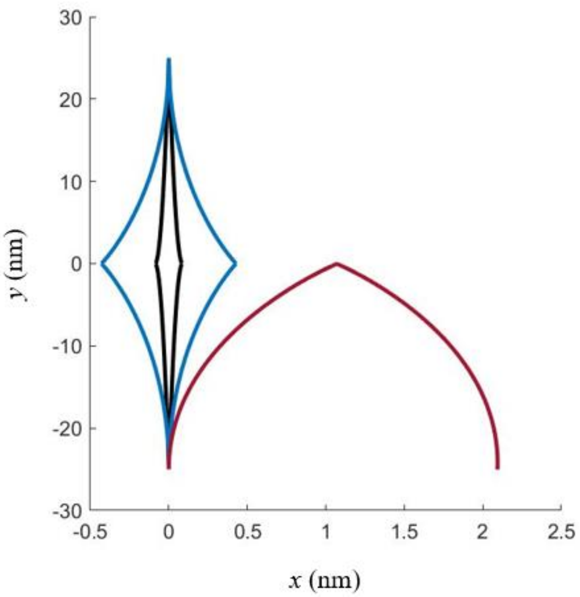

Supplementary Figure 13. Carrier Trajectory Simulation under Different Magnetic Field. Simulation demonstrates the qualitative carrier trajectories in the micro-cavity when magnetic field at 0 T with 0.1° incident angle (black), 0.3 T with 0° incident angle (blue), and 0.5 T with 0° incident angle (red), respectively. x and y are the directions that across the junction and along the junction boundary,

Supplementary Note 15. Simulation of Charge Carrier Trajectory under Finite Magnetic Field

By considering that the carrier density varies linearly inside the microcavity and setting the

carrier density at the cavity boundary as ~ 7×10^{11} cm^{-2}, we model the carrier movement in the cavity in a semi-classical way where the trajectory is integrated by arcs with varying radius described by

$$R_c = \frac{\hbar\sqrt{\pi n}}{eB}.$$

Supplementary Fig. 13 shows the simulated carrier trajectories (with small incident angles) under external magnetic field of 0 T (black), 0.3 T (blue), and 0.5 T (red), as a qualitative demonstration. The implication of magnetic field becomes more relevant closer to the charge neutrality line. As B approaches 0.5 T (red), the effective incident angle becomes large enough that the majority of the carriers are reflected at the charge neutrality line, thus no longer contributing to Veselago interference. The semi-classical simulation qualitatively agrees with our experimental observation.

Quantitative analysis of "critical field" is beyond of the scope and the central thesis of the work. First, multiple interference loops with a distribution simultaneously contribute to the observed the resistance peak, its broadening and eventual disappearance (as a function of B) is smooth, with critical field ill-defined. Moreover, the magnetic field dependence is qualitatively reproduced in multiple devices, but the quantitative details vary from device to device (Supplementary Note 5), depending on realistic experimental variations such as smoothness of resulting PN junction, the specific size and uniformity of atomic strain at the boundary of the cavity, even in the state-of-the-art graphene devices.

Supplementary Note 16. Collimation efficiency of other devices

As shown in Supplementary Fig. 14, we reproduced the trend of suppression in transverse voltage along $4|n_1| = |n_2|$ within a background of high signal. Instead of resistance peaks in the region where $|n_1| > |n_2|$ as in Devices 1 and 2, here we find more complex and finer alternation between resistance peaks and dips, which may be due to the two sets of interferences in Devices 4 and 5.

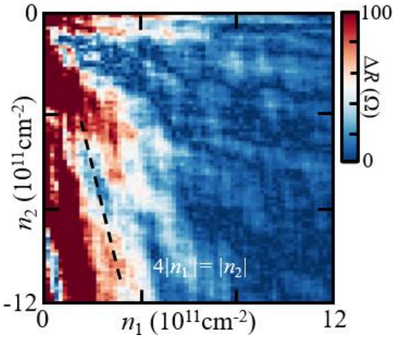

Supplementary Figure 14. Measured transverse voltage of a device similar to Devices 1 and 2, with two transverse voltage probes allowing characterization of collimation efficiency.

Supplementary Note 17. Band structure calculations of strained graphene

To confirm the existence of band gaps in strained graphene as well as achieve a qualitative demonstration of a gap can be opened by strain, we performed first-principles band structure calculations based on Density Functional Theory (DFT) (Supplementary Fig. 15c). DFT calculations were performed using the VASP code[11,12]. Taking the graphene lattice constant to be 2.46 Å, we applied local strain to a two Carbon bonds of a 7x1 monolayer graphene layer along the zigzag direction (see Supplementary Fig. 15a). In Supplementary Fig. 15c, the results were obtained with carbon bond being strained by 17.5%. In the calculation, we used a plane wave energy cutoff of 700 eV, PAW pseudopotentials, and PBE exchange-correlation functional[13]. We

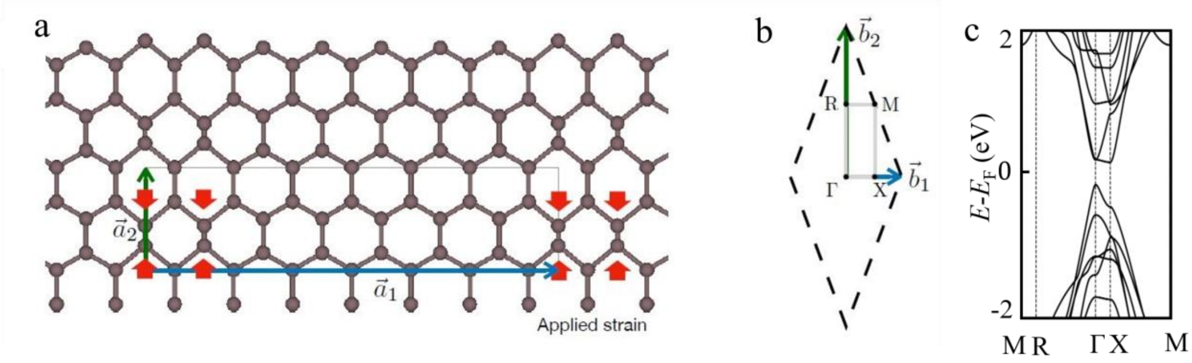

Supplementary Figure 15. Band structure calculations. (a) Geometry of strained monolayer graphene in real space. Red arrows indicated the applied strain. Gray rectangle is the unit cell spanned by the blue and green vectors. (b) Corresponding reciprocal space (black dashed line) spanned by blue and green vectors. The irreducible Brillouin zone is shown in gray. Letters are the high-symmetry points used in Fig. 1d. (c) Band structure of locally-strained monolayer graphene along the high symmetry line shows a band gaps ~ 0.4 eV.

first calculated the charge density self-consistently by allowing the atoms to relax. In the self-consistent calculation, the atomic structures were relaxed until the forces on each atom were below 10^{-6} eV/Å. The Brillouin zone (BZ) was sampled by a 21x31x1 gamma-centered grid. In the band structure calculation, we sampled along the high symmetry line $\mathrm{M} \to \mathrm{R} \to \Gamma \to \mathrm{X} \to \mathrm{M}$ (see Supplementary Fig. 15b), with each line segment being sampled with 100 points. Our results are consistent with Gui *et al.* [14]

The theoretical calculation shows the band structure of strain over a few unit cells, simply because a quantitative calculation for the experimental reality, a smooth strain over 8 nm, is unrealistic. The theoretical band calculation is intended to qualitatively show that atomic strain can results into bandgap. It is not intended to show that the device needs a sharp atomic distortion (over a few unit cells) or a gap as large as 0.4 eV to operate as it designed to be. In fact, a large strain-induced gap will result into an extremely sharp angle-dependence of Klein tunneling rate, rendering effective no carriers with finite incident angle to enter the cavity, let alone Veselago inference. A smooth atomic strain is not only capable of hosting Veselago interference, but actually preferred and an integral part of the experimental design.

References

1. Yang, C.-F. & Hwu, J.-G. Role of fringing field on the electrical characteristics of metal-oxide-semiconductor capacitors with co-planar and edge-removed oxides. *AIP Advances* **6**, 125017 (2016).
2. Farmer, D. B., Perebeinos, V., Lin, Y.-M., Dimitrakopoulos, C. & Avouris, P. Charge trapping and scattering in epitaxial graphene. *Phys. Rev. B* **84**, 205417 (2011).
3. Kim, S. *et al.* Realization of a high mobility dual-gated graphene field-effect transistor with Al2O3 dielectric. *Appl. Phys. Lett.* 4.

4. Bolotin, K. I. Electronic transport in graphene: towards high mobility. in *Graphene* 199–227 (Elsevier, 2014). doi:10.1533/9780857099334.3.199.
5. Wang, K. *et al.* Tunneling Spectroscopy of Quantum Hall States in Bilayer Graphene p − n Junctions. *Phys. Rev. Lett.* **122**, 146801 (2019).
6. Neil W. Ashcroft , N. David Mermin. *Solid State Physics,* CENGAGE Learning (1976).
7. Cheianov, V. V. & Fal'ko, V. I. Selective transmission of Dirac electrons and ballistic magnetoresistance of n − p junctions in graphene. *Phys. Rev. B* **74**, 041403 (2006).
8. Young, A. F. Quantum interference and Klein tunnelling in graphene heterojunctions. *NATURE PHYSICS* **5**, 5 (2009).
9. Milovanović, S. P., Moldovan, D. & Peeters, F. M. Veselago lensing in graphene with a p-n junction: Classical versus quantum effects. *Journal of Applied Physics* **118**, 154308 (2015).
10. Wilmart, Q. *et al.* A Klein-tunneling transistor with ballistic graphene. *2D Mater.* **1**, 011006 (2014).
11. Kresse, G.; Furthmüller, J.; Efficient iterative schemes for ab initio total-energy calculations using a plane-wave basis set. *Physical Review B* **1996,** *54*, 11169.
12. Kresse, G.; Furthmüller, J.; Efficiency of ab-inito total energy calculations for metals and semiconductors using a plane-wave basis set. *Computational Materials Science* **1996,** *6* (15).
13. Perdew, John P., Kieron Burke, and Matthias Ernzerhof. "Generalized gradient approximation made simple." *Physical Review Letters* **77**, no. 18 (1996): 3865.
14. Gui, G., Morgan, D., Booske, J., Zhong, J., & Ma, Z. (2015). Local strain effect on the band gap engineering of graphene by a first-principles study. *Applied Physics Letters*, **106**(5), 053113.
